# QTL-Seq Identifies Extra QTLs and Candidate Genes Controlling High Haploid Induction Rate in Maize

**DOI:** 10.3390/plants15060855

**Published:** 2026-03-10

**Authors:** Kanogporn Khammona, Abil Dermail, Yu-Ru Chen, Wanchana Aesomnuk, Thomas Lübberstedt, Samart Wanchana, Theerayut Toojinda, Siwaret Arikit, Khundej Suriharn, Vinitchan Ruanjaichon

**Affiliations:** 1 Rice Science Center, Kasetsart University, Kamphaeng Saen Campus, Nakhon Pathom 73140, Thailand; kanogporn.k@ku.th (K.K.); siwaret.a@ku.th (S.A.); 2 Department of Agronomy, Faculty of Agriculture, Khon Kaen University, Khon Kaen 40002, Thailand; abildermail@rocketmail.com; 3 Department of Agronomy, Iowa State University, Ames, IA 50011, USA; yuruchen@iastate.edu (Y.-R.C.); thomasl@iastate.edu (T.L.); 4 National Center for Genetic Engineering and Biotechnology (BIOTEC), 113 Thailand Science Park, Pahonyothin Road, Khlong Nueng, Khlong Luang, Pathum Thani 12120, Thailand; waesomnuk@gmail.com (W.A.); samartw2006@gmail.com (S.W.); toojindatheerayut@gmail.com (T.T.); 5 Department of Agronomy, Faculty of Agriculture at Kamphaeng Saen, Kasetsart University, Kamphaeng Saen Campus, Nakhon Pathom 73140, Thailand; 6 Plant Breeding Research Center for Sustainable Agriculture, Faculty of Agriculture, Khon Kaen University, Khon Kaen 40002, Thailand

**Keywords:** maize haploid inducers, QTL-seq analysis, haploid induction rate, doubled haploid technique, maternal haploid induction

## Abstract

Double-haploid (DH) technology is a well-established method for speeding up the development of inbred lines in breeding programs. The major loci *qhir1* and *qhir8* are widely used in marker-assisted selection (MAS) to increase the haploid induction rate (HIR) in maize. However, previous studies have shown that HIR can be unstable within populations, even in the presence of these two loci. To identify novel loci associated with HIR, we performed QTL-seq analysis on 337 S_2_ haploid inducers (*qhir1+/qhir8+*) derived from crossing K8 with BHI306. The population exhibited HIR ranging from 0% to 31.16%. We sequence-bulked DNA from 30 extremely high-HIR lines (15.72–31.16%) and 30 extremely low-HIR lines (0–3.84%), identifying candidate intervals on chromosomes 2 (*qHI2*), 3 (*qHI3*), 6 (*qHI6*), and 8 (*qHI8*). Based on the QTL-seq results, 147 high-confidence SNPs/InDels (R^2^ > 0.3) led to the analysis of 58 genes across three QTLs. We retrieved ten missense mutation SNPs from three genes (*GRMZM2G359746* (*qHI2*), *AC198725.4* (*qHI3*), and *GRMZM2G091276* (*qHI8*)), which are located on chromosomes 2, 3, and 8. Regression analysis of these SNPs showed an R^2^ range of 0.27 to 0.72. The two most highly associated SNPs were located in exon 2 of *GRMZM2G359746* (*qHI2*) and in exon 5 of *GRMZM2G091276* (*qHI8*), respectively. Marker–trait association analysis revealed that lines carrying favorable alleles at both loci, together with *qhir1+* and *qhir8+,* exhibited significantly higher average HIR (12.77%) compared to those with unfavorable alleles (6.66%). These findings provide valuable markers for enhancing maternal haploid inducer breeding programs in maize.

## 1. Introduction

Maize (*Zea mays* L.) is one of the most widely cultivated crops in the world [1]. The top three producing countries are the United States, China, and Brazil, accounting for 32%, 24%, and 10% of the global production, respectively [2]. Maize is primarily grown for livestock feed and fuel ethanol production. Maize growers commonly use F_1_ hybrid seeds because they offer advantages such as higher yields, greater uniformity, and improved lodging resistance [3].

QTL-seq analysis is a widely used and powerful method for mapping quantitative trait loci (QTL). Takagi et al. [4] first reported and published a protocol that successfully identified QTL in rice using recombinant inbred lines (RILs) and F_2_ populations. This method can be applied to any population type to detect genomic regions that have undergone artificial or natural selection. However, QTL-seq has one limitation: it is not suitable for detecting QTL with minor effects because it is not possible to take replicated measurements for each genotype. In this approach, DNA is extracted from two groups with extreme phenotypes within a segregating population, as well as from the parental lines, for whole-genome sequencing. The ΔSNP index is used as the statistical model. First, the k-value is calculated, representing the number of reads with an allele that differs from the reference. Then, the SNP index for the two extreme bulked DNA samples is calculated using the formula:

$$\mathrm{SNP}\text{-}\mathrm{index}=\frac{k1}{n},$$

where n is the total number of reads. Typically, only SNPs with an index greater than 0.3 are retained for further analysis. Then, a sliding window approach is applied to visualize the graphs based on the SNP index. Next, the ΔSNP index is calculated as follows: ΔSNP index = SNP index of the highest bulk–SNP index of the lowest bulk. Finally, QTL are identified in regions with the highest ΔSNP index, assuming that these genomic regions differ significantly between the two bulks. A high ΔSNP index indicates that the genomic regions are representative of the highest bulk or related to phenotypes observed in the uppermost population groups [4,5]. QTL-seq has been widely used in various crops, including capsicum [6], soybean [7], peanuts [8,9], tomatoes [10,11,12], bottle gourds [13,14], pears [15], radishes [16], and rice [9,17,18,19,20,21,22,23,24,25,26,27,28,29,30,31].

The DH technique is an essential tool in commercial maize breeding programs because it is the fastest and most efficient way to generate completely homozygous lines, surpassing conventional inbred development [32]. This process uses maternal haploid inducers to produce pollen for haploid seed development [33,34]. Subsequent chromosome doubling of the haploid genome, followed by self-pollination, yields the final homozygous DH lines [32]. Two critical factors that govern DH efficacy are the haploid induction rate (HIR) and the DH technique. Recently, two major QTL controlling HIR were identified: *qhir1* and *qhir8*. The underlying gene of *qhir1* is one of these genes: *MATRILINEAL* (*MTL*), *ZmPHOSPHOLIPASE-A1* (*ZmPLA1*), or *NOT LIKE DAD* (*NLD*). This gene is characterized by a 4-bp insertion in the fourth exon. In contrast, the *Zea mays DUF679 domain membrane protein* (*ZmDMP*) gene in the *qhir8* region exhibits a single-nucleotide substitution mutation [11,35,36]. The *ZmDMP* mutation alone results in a low basal HIR of approximately 0.15%, but when it is presented with *MTL*, there is a 2–3-fold increase in HIR [11].

Additionally, mutations induced by CRISPR-Cas9 in the *Zea mays PHOSPHOLIPASE D3* (*ZmPLD3*) gene can increase HIR by up to threefold (from 1.19% to 4.13%) when *MTL* is also present [37]. CRISPR-Cas9-induced mutations in the *peroxidase65* (*ZmPOD65*) gene, which belongs to the reactive oxygen species (ROS) class, can lead to HIR ranging from 0.9% to 7.7% [38]. A recent genome-wide association study (GWAS) involving 952 lines, comprising 159 haploid inducers and 793 non-inducers, identified one significant QTL on chromosome 10 and a QTL with a smaller effect on six of the ten chromosomes [39]. Furthermore, a novel *Centromeric Histone H3* (*cenh3*) gene associated with centromere failure caused by *CENH3* dilution during post-meiotic cell divisions preceding gamete formation has been discovered. This dilution increases HIR to approximately 5% when crossing maize heterozygous for a *cenh3* null mutation with wild-type plants to produce haploid progeny [40].

However, in our previous study, we found that HIRs are not stable, even in the presence of *qhir1* and *qhir8*, when using haploid inducer populations in generations F_3_ and F_4_ [41]. To better understand the genetic basis of HIR, we performed a QTL-seq analysis of 337 S_2_ haploid inducer lines derived from a cross between K8, which has low levels of HIR, and the high-HIR inducer line BHI306. This study aims to (i) identify QTL associated with high HIR through QTL-seq analysis and (ii) gain a deeper understanding of the molecular mechanisms underlying haploid induction in maize.

## 2. Results

### 2.1. Phenotyping of the Mapping Population and Selection of Extremely High and Low HIR

**Figure 1:**
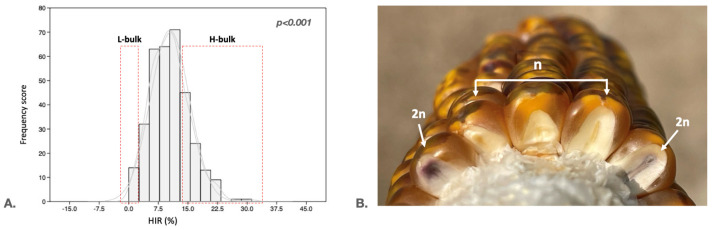
Evaluation of the HIR in 337 S_2_ inducer lines. (**A**). Distribution of HIR in the 337 inducer population. The dashed rectangles indicate the lines selected to generate the H and L bulks. (**B**). The phenotypic appearance of haploid (n) and diploid (2n) seeds was evaluated using the *R1-nj* biomarker.

We developed a population of 337 S_2_ haploid inducers by crossing K8 (*qhir1-/qhir8-*, low HIR, tropical) with BHI306 (*qhir1+/qhir8+*, 10–15% HIR, temperate). Nineteen superior S_1_ lines (*qhir1+/qhir8+*) were selected and assigned to two groups (Group A: ten families; Group B: nine families) based on genetic diversity to maximize recombination, yielding 337 S_2_ lines. We employed this intercrossing strategy to maintain genetic variation at other loci while fixing the major HIR QTLs. This facilitates the identification of additional minor-effect loci. This revealed that the HIR frequency distribution histogram is skewed toward low HIR values in populations (Figure 1). To identify the genomic region associated with HIR, we performed QTL-seq analysis on haploid inducers derived from a cross between BHI306 (the male parent) and a tropical inducer (K8). A total of 337 S_2_ haploid inducer lines were used in the study (Table S1). To ensure the quality and robustness of phenotyping, we exploited the differences between haploid (n) and diploid (2n) individuals at the seedling stage to minimize errors.

### 2.2. Whole-Genome Resequencing, Sequence Processing, and Variant Calling

Thirty extremely high and low HIRs were selected from each side. Their entire genome was captured using the BGISEQ-100 platform (Shenzhen, China). The cleaned reads from BHI306 and K8 were as follows: 186.83 and 140.32 million, respectively. This corresponded to a genome coverage of approximately 12.28- and 9.39-fold, respectively, given an estimated maize genome size of 2058 megabase pairs (Mb).

A total of 1521 million and 1415 million cleaned reads were obtained from the 60 haploid inducer samples. The sequences of the 30 haploid inducers with high HIR were grouped together and referred to as high bulk (H bulk), while the sequences of the 30 haploid inducers with low HIR were grouped together and referred to as low bulk (L bulk). Based on read alignment to the B73 reference genome, the proportion of aligned reads was as follows: BHI306 (89.29%), K8 (87.58%), H bulk (99.95%), and L bulk (99.98%) (Table 1).

### 2.3. QTL-Seq Analysis and Marker Validation in the Maize Haploid Inducer Population

**Figure 2:**
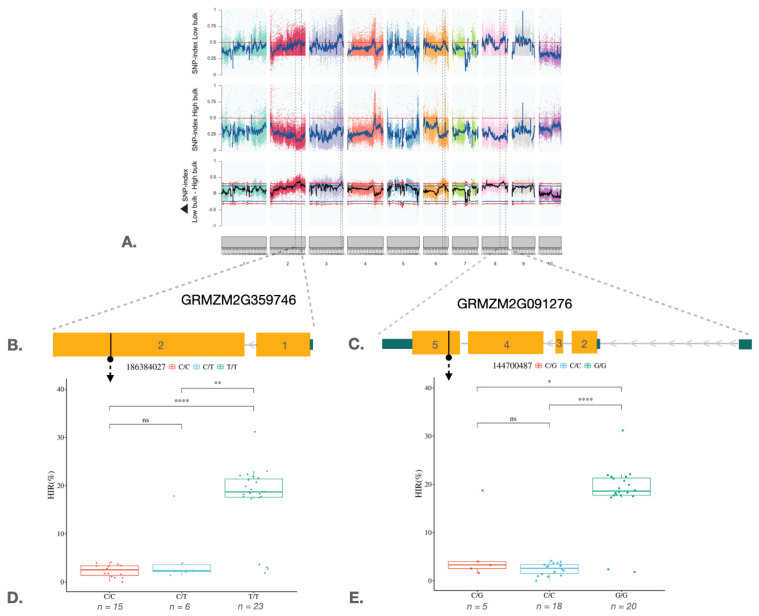
Plots of the SNP index for the H and L bulk and the ΔSNP index across ten maize chromosomes. (**A**). The graphs depict the SNP index for the L and H bulk and the Δ SNP index from QTL-seq analysis. The blue and red line pairs represent 95% and 99% confidence intervals, respectively, and the pink vertical bar represents the peak SNP in the candidate region. (**B**). Structure of the *WEB1* gene (*GRMZM2G359746*). The yellow box represents an exon. (**C**). Structure of the *JAR1a* gene (*GRMZM2G091276*). The yellow box represents an exon. (**D**) Box plots of SNP position 186384027 belonging to the *WEB1* gene. (**E**) Box plots of SNP position 144700487 belonging to the *JAR1a* gene. (*, **, and **** represent *p*-values of less than 0.05, 0.01, and 0.0001, respectively).

QTL-seq analysis was performed using the QTL-seq pipeline [42]. The SNP variants used in this analysis were common SNPs identified in both the H and L bulks based on read mapping against the BHI306 parental genome. Initially, 90,626 SNPs and 33,595 InDels were identified in the two bulks with a read support criterion of at least seven reads (Table 2). We calculated the ∆SNP index by subtracting the SNP index values in the H bulk from those in the L bulk. This calculation was based on moving windows that averaged SNP index values within 1 Mb regions with 100 kb increments. Then, we plotted the ∆SNP index across the ten maize chromosomes to identify genomic regions associated with high HIR (Figure 2A). As a result, four QTLs were located on chromosomes 2, 3, 6, and 8, where the average ∆SNP index exceeded the 99% confidence interval with values ranging from 0.31 to 0.32 (Table 3). We evaluated the R^2^ values for 5224 SNPs within the four QTLs including qHI2 (∆SNP index = 0.39), qHI3 (∆SNP index = 0.40), qHI6 (∆SNP index = 0.35), and qHI8 (∆SNP index = 0.38) and found that 147 SNPs with R^2^ values greater than 0.3 were associated with qHI2, qHI3, and qHI8 and six candidate genes: *GRMZM2G140156*, *GRMZM2G359746*, *GRMZM2G440943*, *AC198725.4*, *GRMZM2G091276*, and *GRMZM2G134738* (Table 3). Ten of these SNPs caused missense mutations in three genes: *GRMZM2G359746*, *AC198725.4*, and *GRMZM2G091276*. These mutations occurred on chromosomes 2, 3, and 8 (Table 4). Two SNPs with R^2^ values greater than 0.5, including the first SNP located on chromosome 2 in the *WEB1* gene (GRMZM2G359746) at exon 2 and the second SNP located on chromosome 8 in the *JAR1a* gene (*GRMZM2G091276*) at exon 5, were selected for Kompetitive Allele-Specific PCR (KASP) marker development (Figure 2B–E).

**Figure 3:**
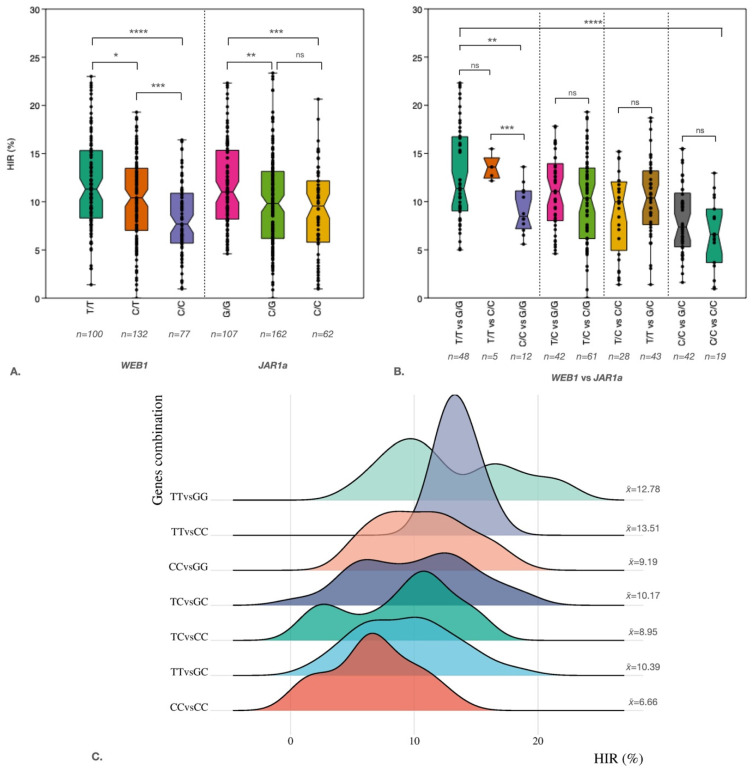
WEB1_2_186384027 and JAR1a_8_144700487 KASP marker validation. (**A**). Box plot showing the genotype–phenotype correlations of HIR and the WEB1_2_186384027 (n = 309) and JAR1a_8_144700487 (n = 331) markers, respectively, in the S_2_ population. (**B**). Box plot showing combinations of the two markers. (**C**). A ridgeline plot showing the density of each combination of the two markers. (*, **, ***, and **** represent *p*-values of less than 0.05, 0.01, 0.001, and 0.0001, respectively).

To test the relationship between genes and HIR, two KASP markers were developed from the *WEB1* and *JAR1a* genes identified in the QTL-seq analysis. These markers were then used to analyze the association between markers and traits. For the *WEB1* gene, a marker was designed from SNP position 186384027 (WEB1_2_186384027) on chromosome 2 of the maize genome reference version 2 (V.2). A nucleotide substitution from T to C in exon 2 resulted in an amino acid change from arginine to glutamine (Table 4). The *JAR1a* marker was designed from the SNP position 144700487 (JAR1a_8_144700487) on chromosome 8 (V.2). A nucleotide substitution from C to G on exon 5 led to an amino acid change from glycine (G) to arginine (R) and was used to genotype the S_2_ population (Table 4). Marker–trait association results revealed a highly significant association between both markers and the phenotypes (*p*-value < 0.001) (Figure 3A,B). The phenotypic variance explained (PVE) by markers WEB1_2_186384027 and JAR1a_8_144700487 was 8% and 3%, respectively, in the entire population (Table 5). Additionally, we found that the presence of the *WEB1* and *JAR1a* genes in homozygous TT and GG forms, respectively, leads to HIR of up to 12.78%. However, HIR is only 6.66% when the genotype is homozygous CC and CC, respectively (Figure 3C).

## 3. Discussion

In this study, a total of 6 annotated candidate genes were associated with SNPs that exhibited an R^2^ value ranging from 0.27 to 0.72. These candidate genes were mainly located on chromosomes 2, 3, and 8. According to the MaizeGDB database, two genes of particular interest are *GRMZM2G359746* (*WEAK CHLOROPLAST MOVEMENT UNDER BLUE LIGHT 1*), which is located on chromosome 2 and is highly related to chloroplast photo relocation movements under blue light. With regard to *WEB1* localization in the cytosol, Kodama et al. [43] reported that the *WEB1* mutation in *Arabidopsis* caused the chloroplast avoidance movement to occur more slowly than that of WT under strong blue light conditions due to the regulations of cp-actin filaments being impaired in the mutant [43]. Although the strong blue light can induce reactive oxygen species (ROS), there is no evidence that *WEB1* is related to ROS. Majumdar and Kar [44] reported that chloroplast ROS generation occurs via thylakoid membrane-located large, multi-subunit oxidoreductase protein complexes, namely photosystem I and II (PS I and PS II). Other authors also found that in strong light conditions, the rates of energy transfer and electron (e^−^) transport through the photosynthetic e^−^ transport chain (ETC) are much slower than light energy harvesting [45,46,47,48]. O_2_˙^−^ is produced significantly at the reducing side of photosystem I (PS I), where molecular O_2_ competes with NADP^+^ for e^−^ from PS I, acting as the terminal e^−^ acceptor and producing O_2_˙^−^ by the Mehler reaction [48,49], whereas H_2_O_2_ may originate from incomplete oxidation of H_2_O or a one-electron reduction of O_2_˙^−^ [50,51]. From this point, we believe that the *WEB1* gene may be involved in the avoidance movement of chloroplasts, resulting in ROS in maize leaves. This leads to damaged pollen grains and haploid seeds. The associated SNP, denoted as WEB1_2_186384027, is located on chromosome 2 within exon 2 of the *WEB1* gene, with an R^2^ value of 0.58.

The *GRMZM2G091276* (*Jasmonate-resistant 1*) gene is located on chromosome 8 and is highly related to late stamen development. Song et al. [52] revealed that Arabidopsis *jasmonate-aminosynthetase/jasmonate-resistant 1* (*JAR1a*) belongs to the *jasmonic acid* (*JA*) biosynthesis pathway as one of the structural genes. *JA* has been shown to play a role in stamen development, root growth, trichome formation, leaf senescence, anthocyanin accumulation, and defense against insects and pathogens [53,54,55,56,57,58]. *JAR1a* has been reported to convert *acyl-CoA oxidase* (*ACX*) into *jasmonoyl-L-isoleucine* (*JA-Ile*), which is the bioactive form of *JA* that is perceived by *COI1*. *COI1* then recruits JAZ proteins for ubiquitination and degradation via the 26S proteasome. Degradation of the JAZ proteins releases the MYB21, MYB24, and MYB57 downstream factors to regulate late stamen development. Mutations in genes that encode JA biosynthetic enzymes result in filament elongation failure, delayed anther dehiscence, and unviable pollen grains at floral stage 13 [59,60,61]. From this, we believe that the *JAR1a* gene may be involved in pollen grain development. This leads to immature pollen grains and haploid seeds. The associated single-nucleotide polymorphism (SNP), denoted as JAR1a_8_144700487, is located on chromosome 8 within exon 5 of the *JAR1a* gene, with an R^2^ value of 0.72.

Of the markers evaluated in the entire S_2_ population, the percentage of variance explained (PVE) was found to be low. However, we found that the average HIR increased in both gene mutations, especially in the *WEB1* gene. The maximum HIR increased to 23.01%, compared to 16.42% in the wild type (WT). The *JAR1a* gene mutation maintains a minimum HIR of 4.6%, compared to 0.95% in WT (Figure 3A). We believe that this finding will enable breeders to increase the HIR selection index in the haploid inducer breeding program in the future.

Key players in maternal haploid induction include the membrane protein *DOMAIN OF UNKNOWN FUNCTION 679* (*DMP*), which has been shown to play a role in gamete fusion during double fertilization [62,63]. The *MTL/ZmPLA1/NLD* gene, which encodes a pollen-specific phospholipase, has also been associated with the haploid induction process in maize. It regulates the formation and development of maize pollen as well as pollen tube elongation [64,65,66]. However, the *WEB1/PMI2* complex was found to suppress the chloroplast accumulation response control [67], and the *JAR1a* gene was found to be related to late stamen development [52]. However, further validation is needed to confirm the involvement of these genes in haploid induction. Nevertheless, we found that these two genes could improve HIR by an average of 12.78% (*qhir1+/qhir8+/web1+/jar1a+*) from 6.66% (*qhir1+/qhir8+*). However, stability remains low due to the wide range of HIR in the population with *qhir1+/qhir8+/web1+/jar1a+* (4.6–23.01%). This suggests that other genes or QTLs may play a role in this stability.

## 4. Materials and Methods

### 4.1. Plant Materials and HIR Evaluation

**Figure 4:**
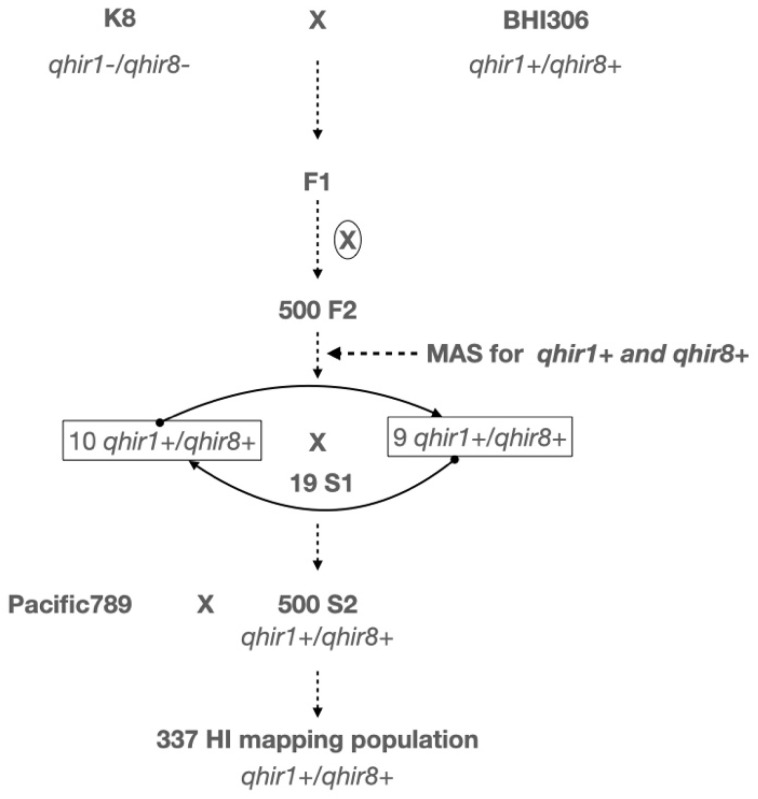
The schematic of the S_2_ population development.

A population of 337 S_2_ haploid inducers was developed from crossing K8 (*qhir1-/qhir8-_* homozygous dominant, low HIR, tropical) x BHI306 (*qhir1+/qhir8+_* homozygous recessive, HIR of 10–15%, temperate) (Figure 4). BHI306, developed by Iowa State University’s Doubled Haploid Facility, carries the *R1-nj* and *Pl-1* markers for haploid identification. F_1_ plants were self-pollinated to produce F_2_ progeny. Using *qhir1* and *qhir8*-specific markers [41], we genotyped the F_2_ population and selected 19 individuals with *qhir1+/qhir8+* genotypes named as S_1_. Based on preliminary HIR screening and marker analysis, 19 superior S_1_ were selected and assigned to two groups (Group A: 10 families; Group B: 9 families) based on genetic diversity to maximize recombination. The pollen from each group was bulked for reciprocal intercrosses between groups, generating 337 S_2_ lines, all confirmed as *qhir1+/qhir8+* through marker-assisted selection. This intercrossing strategy was employed to maintain genetic variation at other loci while fixing the major HIR QTLs, thereby facilitating the identification of additional minor-effect loci.

**Figure 5:**
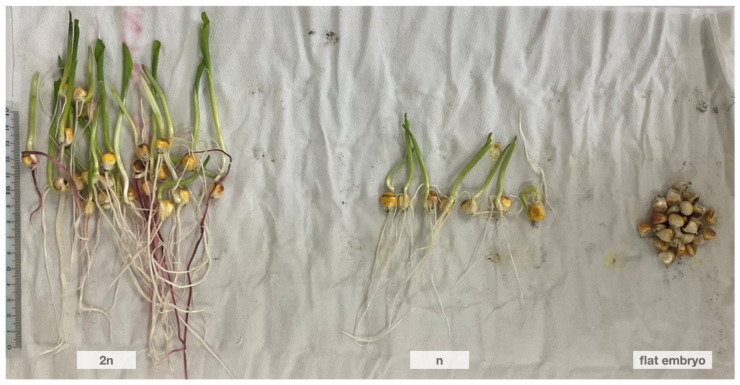
The innate difference between diploid and haploid young seedlings at five days after germination.

HIR was evaluated by crossing each S_2_ inducer (as male) to the commercial hybrid Pacific789 (as female). Due to variable flowering times, the donor was planted four times at 5-day intervals. Each inducer pollinated one ear without replication. Standard precautions (bagging, detasseling) prevented contamination. Haploid seeds were identified using the *R1-nj* marker (purple crown endosperm, colorless embryo vs. diploid with purple in both tissues). HIR was calculated as follows:

$$\mathrm{HIR}\;(\%)=\frac{\mathrm{seed}\mathrm{number}\mathrm{of}\mathrm{putative}\mathrm{haploid}}{\mathrm{seed}\mathrm{set}}\times 100$$

where the seed set represents the total seed number of haploid seeds, diploid seeds, and the seeds without the *R1-nj* marker. Haploid identification was validated using the method described by Dermail et al. [68] that uses seedling morphology (radicle and coleoptile length) to predict the size of haploid seedlings relative to diploid seedlings. According to this method, haploid seedlings will be approximately half the size of diploid seedlings and have white roots (Figure 5).

### 4.2. Selection of Plants with Extremely High and Low Haploid Induction Rates, DNA Isolation and Whole-Genome Resequencing

A total of 337 S_2_ haploid inducers were identified by the HIR using the *R1-nj* biomarker and innate differences (Table S1). The groups with the highest and lowest HIRs were selected for QTL-seq analysis. High-quality genomic DNA was extracted from the leaves of 30 high- and 30 low-HIR S_2_ haploid inducer plants (Table S2), as well as from the BHI306 and K8 parental lines, using a DNeasy Plant Mini Kit (Qiagen, Germany). The DNA samples were sent to BGI (BGI-Shenzhen, China) for whole-genome sequencing using the BGISEQ-100 platform.

### 4.3. Processing of Sequencing Data and QTL-Seq Analysis

Raw reads were trimmed and filtered using Trimmomatic (v. 0.40) to remove low-quality sequences [22]. Of the 30 samples in the high HIR group, 1.521 billion clean reads were randomly selected and pooled to create the highest bulk (H bulk). Similarly, 1.415 billion clean reads were randomly selected from each of the 30 samples in the lowest HIR group and pooled as the low bulk (L bulk). These two pools were then used to perform QTL-seq analysis following the pipeline described by Takagi et al. [4] and Sugihara et al. [42].

To identify single-nucleotide polymorphisms (SNPs) and insertion-deletion variants (indels), the short reads from both bulks were aligned to the BHI306 reference genome. The SNP index was calculated at all identified SNP positions for both the H and L bulks. SNP positions with an index value of less than 0.3 and a read depth of less than seven were excluded from both bulks because they may be spurious SNPs resulting from sequencing and/or alignment errors [4]. The preprocessed reads from each sample were also aligned to the B73 reference genome (PRJNA10769) using the GATK best-practices pipeline to detect SNP and indel variants [68]. The result of single-sequencing and SNP/indel data was used for allelic variation analysis and candidate gene identification.

### 4.4. Design a Marker for HIR Validation

Two KASP markers were developed from the *WEB1* and *JAR1a* genes identified by QTL-seq analysis and used to analyze marker–trait associations. The *WEB1* marker was designed from the SNP position 186384027 on chromosome 2 of the maize genome reference version 2 (V.2), and the *JAR1a* marker was designed from the SNP position 144700487 on chromosome 8 (V.2) (Table 4). The KASP conditions were set as follows: an initial temperature of 94 °C for 5 min, followed by 10 cycles of 94 °C for 20 s and 61 °C for 60 s (touchdown to 61 °C with a decrease of 0.6 °C per cycle), followed by 27 cycles of 94 °C for 20 s, 55 °C for 30 s, and a rest period of 1 min at 37 °C. After amplification, the fluorescence signals of the final PCR products were read using a QuantStudio 6 Flex Real-Time PCR System (Thermo Fisher Scientific, Waltham, MA, USA) [22]. For marker–trait association analysis, genotype data were obtained by genotyping 337 S2 lines (Table S1). Marker–trait association analysis was performed using a simple regression method and the lm() function in R (version 4.5.2, http://www.r-project.org/).

## 5. Conclusions

This study evaluated a total of 337 S_2_ haploid inducers with extremely high and low haploid induction rates (HIRs) using the QTL-seq technique. Candidate intervals associated with HIR were located on chromosomes 2, 3, 6, and 8. Based on the annotation results, 147 single-nucleotide polymorphisms (SNPs)/insertion-deletion polymorphisms (InDels) were retrieved from 58 candidate genes. Of these, 10 missense mutation SNPs exhibited an R^2^ value ranging from 0.27 to 0.72. According to MaizeGDB data, two genes potentially related to HIR were identified: *WEB1* (*GRMZM2G359746*) and *JAR1a* (*GRMZM2G091276*). Marker–trait association analysis showed that *WEB1* and *JAR1a* can increase the average HIR in the S_2_ population. This indicates that these genes may be significantly involved in the HIR trait in maize. In the future, it may be necessary to identify the role of these two genes in haploid induction to better understand their mechanisms and improve HIR in maize maternal haploid inducers.

## Figures and Tables

**Table 1:** Statistical analysis of sample sequencing data evaluation.

Sample	Cleaned Reads (Million)	Cleaned Base (Gb)	Alignment (%)	Average Depth Coverage (x)
BHI306	186.83	27.52	89.29	12.28
K8	140.32	20.68	87.58	9.39
Highest HIR bulk	1521.00	229.00	99.95	107.00
Lowest HIR bulk	1415.00	212.00	99.98	99.37

**Table 2:** Statistical analysis of SNP detection and annotation results.

Length (bp)	All Variants (Read Depths > 7)	
	SNPs	InDels
301,354,135	10,615	4200
237,068,873	11,149	4100
232,140,174	16,807	6170
241,473,504	10,476	3692
217,872,852	6239	2722
169,174,353	9796	3510
176,764,762	5623	1663
175,793,759	8146	3273
156,750,706	7519	2632
150,189,435	4256	1633
**2,058,582,553**	**90,626**	**33,595**

**Table 3:** Summary of the genomic region associated with the HIR in maize.

QTL	Chr.	QTL Region	Mb	Confidence Interval (99%)	Δ (SNP Index)	No. of SNP/InDel	R^2^ > 0.3	No. of Genes	Candidate Genes
qHI2	2	170,263,374–210,388,889	40.12	0.31	0.39	1777	69	28	*GRMZM2G140156_RID-transcription factor 2*, *GRMZM2G359746_WEB1 gene_WEAK CHLOROPLAST MOVEMENT UNDER BLUE LIGHT 1*
qHI3	3	213,006,045–223,082,649	10.08	0.32	0.40	1254	35	15	*GRMZM2G440943_Helicase/SANT-associated DNA binding protein*, *AC198725.4_FG009_WRKY DNA-binding protein 28*
qHI6	6	130,180,223–145,992,397	15.81	0.31	0.35	473	2	-	-
qHI8	8	120,056,063–159,995,947	38.94	0.31	0.38	1720	41	15	*GRMZM2G091276_JAR1a_Jasmonate-resistant 1*, *GRMZM2G134738_Cytochrome c oxidase subunit 5b-3 mitochondrial (ZmCOX5b-3)*

**Table 4:** Candidate SNPs of each chromosome region.

SNP Position (V.2)	Chr.	R^2^	*p*-Value	n	Gene	Variation	Mutation	Exon	Amino Acid Change
186384027	2	0.58	1.04 × 10^−9^	42	*GRMZM2G359746*	T/C	missense	2	R -> Q
186384242	2	0.28	1.31 × 10^−4^	42	*GRMZM2G359746*	T/G	missense	2	Q -> H
186384636	2	0.43	5.14 × 10^−7^	43	*GRMZM2G359746*	T/G	missense	2	D -> A
217212966	3	0.34	1.37 × 10^−5^	43	*AC198725.4*	T/G	missense	3	D -> P
217212967	3	0.34	1.37 × 10^−5^	43	*AC198725.4*	C/G	missense	3	D -> P
217214782	3	0.27	1.31 × 10^−4^	44	*AC198725.4*	T/G	missense	1	T -> P
144700162	8	0.27	1.57 × 10^−4^	43	*GRMZM2G091276*	C/T	missense	5	G -> E
144700252	8	0.31	6.44 × 10^−5^	41	*GRMZM2G091276*	C/T	missense	5	G -> D
144700393	8	0.5	2.98 × 10^−8^	44	*GRMZM2G091276*	C/T	missense	5	R -> H
144700487	8	0.72	3.47 × 10^−13^	41	*GRMZM2G091276*	G/C	missense	5	G -> R

**Table 5:** Marker–trait association analysis of the two KASPs.

Marker	Range of HIR	Average	SNP	n	R^2^	*p*-Value	PVE
WEB1_2_186384027	1.39–23.01	11.96	T/T	309	0.08	4.39 × 10^−7^	8
	0–19.3	10.36	T/C				
	0.95–16.42	8.19	C/C				
JAR1a_8_ 144700487	4.6–22.32	11.67	G/G	331	0.03	1.82 × 10^−3^	3
	0–23.36	10.02	G/C				
	0.95–20.65	9.02	C/C				

## Data Availability

The datasets generated and analyzed during the current study are available in the NCBI repository, https://dataview.ncbi.nlm.nih.gov/object/PRJNA1403211 (accessed on 15 January 2026), with the accession number PRJNA1403211.

## Supplementary Materials

The following supporting information can be downloaded at: https://www.mdpi.com/article/10.3390/plants15060855/s1, Table S1: The haploid induction rate of the haploid inducer populations used in the study; Table S2: Whole-genome resequencing data of selected haploid inducers with high and low haploid induction rates; Table S3: The sequence of two KASP markers belonging to *WEB1* and *JAR1a* genes.

## Abbreviations

The following abbreviations are used in this manuscript:

DH	Double-Haploid
MAS	Marker-Assisted Selection
HIR	Haploid Induction Rate
QTL	Quantitative Trait Loci
SNP	Single Nucleotide Polymorphism
*MTL*	*MATRILINEAL*
*ZmPLA1*	*Zea mays PHOSPHOLIPASE-A1*
*NLD*	*NOT LIKE DAD*
*ZmDMP*	*Zea mays DUF679 domain membrane protein*
*ZmPLD3*	*Zea mays PHOSPHOLIPASE D3*
*ZmPOD65*	*Zea mays Peroxidase65*
ROS	Reactive Oxygen Species
GWAS	Genome-Wide Association
*CENH3*	*Centromeric Histone H3*
*R1-nj*	*R1-Navajo*
*WEB1*	*WEAK CHLOROPLAST MOVEMENT UNDER BLUE LIGHT 1*
*JAR1a*	*Jasmonate-resistant 1*
PS I	photosystem I
PS II	photosystem II
JA	Jasmonic Acid
*ACX*	*Acyl-CoA Oxidase*
*JA-Ile*	*Jasmonoyl-L-Isoleucine*
PVE	Percentage of Variance
